# Stress, resilience, and moral distress among health care providers in oncology during the COVID-19 pandemic

**DOI:** 10.3389/fpubh.2023.1288483

**Published:** 2023-12-19

**Authors:** Waleed Alrjoub, Ghadeer Alarjeh, Khawlah Ammar, Abedalrahman Shamieh, Richard Harding, Christopher Booth, Richard Sullivan, Majeda Al-ruzzieh, Asem Mansour, Omar Shamieh

**Affiliations:** ^1^Centre for Palliative and Cancer Care in Conflict (CPCCC), King Hussein Cancer Centre (KHCC), Amman, Jordan; ^2^Centre of Research Shared Resources, King Hussein Cancer Centre (KHCC), Amman, Jordan; ^3^Faculty of Medicine, The University of Jordan, Amman, Jordan; ^4^Florence Nightingale Faculty of Nursing, Midwifery and Palliative Care, King’s College London, Cicely Saunders Institute, London, United Kingdom; ^5^Faculty of Health Sciences, Cancer Care and Epidemiology, Queen's University, Kingston, ON, Canada; ^6^Institute of Cancer Policy, King’s College London, London, United Kingdom; ^7^Nursing Department, King Hussein Cancer Center, Amman, Jordan; ^8^Director General’s Office, King Hussein Cancer Center, Amman, Jordan; ^9^Department of Palliative Care, King Hussein Cancer Centre (KHCC), Amman, Jordan

**Keywords:** coronavirus pandemic, stress levels, resilience, oncology healthcare professionals, Perceived Stress Scale (PSS), Connor-Davidson Resilience Scale (CD-RSIC), Moral Distress Thermometer (MDT), Jordan

## Abstract

**Background:**

The coronavirus pandemic has potential implications for stress levels and resilience among oncology healthcare professionals (HCPs). This study aims to assess perceived stress, resilience, and moral distress levels among oncology HCPs in Jordan during the pandemic and identify associated risk factors.

**Methods:**

An online cross-sectional survey was conducted among oncology HCPs in Jordan using three validated tools: Perceived Stress Scale (PSS), Connor-Davidson Resilience Scale (CD-RSIC), and Moral Distress Thermometer (MDT). Seven items were used to assess sources of stress.

**Results:**

A total of 965 participants enrolled with a 74% response rate. The participants’ ages ranged from 20 to 74 (mean = 32.74, SD = 5.197), with 79.1% males, 45.1% were physicians, 32.6% were public hospital workers, 57.1% were married, and 56.6% had children below 18 years. Findings indicated moderate perceived stress (Mean = 15.87, SD = 5.861), low resilience (Mean = 29.18, SD = 5.197), and high moral distress (Mean = 4.72, SD = 2.564). Females, unmarried individuals, and younger age groups exhibited higher PSS (*p* = 0.009, *p* < 0.001, and P<0.001) and lower resilience (*p* = 0.024, *p* = 0.034, and *p* = 0.001). Not having children below 18 years correlated with higher perceived stress (*P* < 0.001). In linear regression analysis, age and gender emerged as significant predictors of both perceived stress and resilience. Female participants reported stress related to the risk of contracting COVID-19 (*p* = 0.001), transmitting it to others (*p* = 0.017), social isolation (*P* < 0.001), and having children at home due to school closures (*p* = 0.000). A cohort of 239 participants repeated the survey within a two-month interval, revealed a statistically significant decrease in the CD-RISC scores (*p* < 0.001).

**Conclusion:**

Oncology HCPs in Jordan experienced moderate stress, high moral distress, and poor resilience during the COVID-19 pandemic. These factors may negatively affect the quality of oncology care. Urgent measures are necessary to support HCPs in coping with unforeseen circumstances in the future.

## 1 Introduction

### 1.1 Overview of the COVID-19 situation in Jordan

Jordan, which was recently downgraded as a lower-middle-income country at the close of 2022 (1), boasted a population of 11,285,869 (2). The demographic landscape in Jordan skews towards a predominantly youthful population. In 2021, approximately 71% of the inhabitants fell within the age bracket of 15–65; while a 3.7% were aged 65 and older (3). Jordan’s experience with COVID-19 has seen several waves and government responses. The first case emerged in March 2020, with initial success in containment. However, by October 2020, the first wave hit, peaking in November with over 26,000 cases and 468 deaths (4). The virus was initially contained in refugee camps, with a high recovery rate among affected refugees (5). Recovery began in May 2021, with low daily cases until November when a third wave occurred, peaking in December 2021 with 34,735 cases and 226 deaths. Despite these waves (4), intensive care unit capacity remained manageable (6).

In January 2022, Jordan faced a fourth wave, with a significant portion linked to the Omicron variant. By February 2022, Jordan reported 1,490,473 cases and 13,532 deaths (4), but ICU and ventilator occupancy remained below alarming levels (6).

Jordan’s response to COVID-19 has been characterized by proactive measures, vaccination campaigns, and policies aimed at restoring normalcy while managing the evolving challenges of the pandemic. Early on, safety measures were implemented, including quarantine, lockdowns, and curfews (7, 8). A vaccination campaign began in January 2021, prioritizing high-risk groups (9). By June 2021, over 2 million adults received the first dose, with additional funding from international partners to accelerate vaccination efforts, including refugees (10).

### 1.2 Understanding COVID-19 impact on healthcare providers

Healthcare providers (HCPs) worldwide have faced numerous challenges and stressors in response to the COVID-19 pandemic since its initial outbreak in China in December 2019 (11, 12). These challenges and stressors are often related to patient care, including limited healthcare resources, difficulties in providing the usual level of care, and patients being isolated without family support. Additionally, HCPs may experience work-related stressors such as long working hours, frequent policy and guideline changes related to COVID-19, uncertainty regarding protocols, and fear of infection, especially when there is insufficient protective equipment. Furthermore, HCPs may encounter additional challenges due to social isolation, lockdowns, and the need to care for their own children at home due to school closures (11–13).

The specific challenges faced by HCPs can vary depending on their involvement in the care of COVID-19 patients. For instance, frontline and intensive care unit HCPs may experience higher levels of stress and challenges compared to others (14, 15). Moreover, HCPs involved in cancer care may face additional and significant challenges that are compounded by pandemic-related issues such as medication and HCP shortages. Healthcare systems need to carefully balance the benefits of providing cancer treatment against the risk of COVID-19 transmission in the hospital environment, taking into account the vulnerability of cancer patients with compromised immunity (16).

Various studies have examined stress levels and mental well-being among HCPs in stable environments using different assessment tools. For example, Abraham et al. (17) assessed stressors, morale, and coping mechanisms among staff working in emergency units. Magnavita et al. (18) examined mental and physical well-being in cancer units using the needs questionnaire and positivity scale. While several studies have explored stressors in rapidly evolving environments like COVID-19, William and Lancee (19) found that HCPs reported long-term stress during the SARS outbreak but their mental status remained unaffected. These studies have contributed to the development of strategies to enhance resilience and reduce stress by promoting safety measures, comfort, and social connections (20).

Marjanovic et al. (20) conducted an online survey among 333 nurses to assess the association of the working environment with psychosocial variables. The study focused on predictors of coping attitudes, emotional exhaustion, and anger using validated tools such as the State–Trait Anger Expression Inventory and the Maslach Burnout Inventory. Results showed that lower emotional exhaustion levels were predicted by higher institutional support, less contact with SARS patients, and shorter quarantine times. Maunder et al. (21) surveyed staff members from 13 hospitals in Toronto, Canada, to assess the impact of SARS on their psychological and occupational well-being 13–26 months after the outbreak. The study revealed higher levels of stress and burnout among staff working in SARS treatment hospitals, reduced working hours, and stress-related behavioral consequences.

In their study, Motahedi et al. (22) underscored the significance of addressing the mental health challenges encountered by HCPs during the COVID-19 pandemic. They conducted an investigation involving 140 HCPs in an urban region of Iran. The findings revealed the presence of moderate anxiety levels alongside concurrent moderate to severe depression among the HCPs. Notably, gender, a history of COVID-19-related quarantine, and previous exposure to COVID-19 were identified as factors associated with elevated levels of both anxiety and depression. Given that working in institutions treating COVID-19 patients may expose HCPs to stress and mental health issues, it is important to identify potential stressors and challenges specific to oncology HCPs in Jordan. Tracking changes over time can provide preliminary baseline data to inform future policies and studies, particularly considering the scarcity of research in this area in Jordan. The objectives of this survey are: (1) to describe the impact of COVID-19 on oncology HCPs in Jordan by assessing self-reported perceived stress levels, resilience, and moral distress, (2) to compare these findings within different HCP groups (e.g., nurses vs. doctors, ambulatory vs. in-patient care), and (3) to conduct longitudinal assessments of these mental wellness dimensions over time as the COVID-19 environment evolves.

## 2 Materials and methods

### 2.1 Study design and participants

The study employed a cross-sectional survey with a longitudinal assessment to quantify perceived stress, its sources, resilience, and moral distress among oncology healthcare professionals (HCPs) in Jordan. Data collection was conducted using the Survey Monkey platform from November 9, 2020, to April 28, 2021. The study included frontline HCPs (e.g., doctors, nurses, pharmacists, psychosocial workers) who worked full-time or part-time during the pandemic in COVID-19-designated hospitals in Jordan, encompassing in-patient, ambulatory, and administrative care settings. HCPs who did not work in COVID-19-designated hospitals, did not work at least part-time during the pandemic, or were on vacation were excluded from the study.

### 2.2 Data collection

Data was collected through a structured survey instruments that incorporated both validated psychological scales and demographic information. The data collection process aimed to capture the experiences and mental well-being of oncology HCPs in Jordan during the COVID-19 pandemic.

### 2.3 Data collection instruments

#### 2.3.1 Perceived Stress Scale (PSS-10)

The PSS is a validated self-reported tool used to measure stress levels in individuals who perceive their lives to be unpredictable, uncontrollable, and burdensome over the past month (23). Originally introduced in 1983 by Cohen and colleagues (23), where the PSS initially comprised 14 items. Participants are required to rate the frequency of their experiences of emotions and thoughts associated with life events and situations over the preceding month using a five-point scale, ranging from (0) “Never” to (4) “Very Often.” The cumulative score derived from the PSS serves as a comprehensive measure of perceived stress. A short version of the PSS (PSS-10) was developed by Cohen and Williamson (24) in 1988. This adaptation was created by excluding four items with the lowest factor loadings from the original scale. The psychometric characteristics of the PSS-10 were initially assessed in a large national sample in the United States. The PSS-10 demonstrated satisfactory internal consistency reliability (α = 0.78). Furthermore, moderate concurrent criterion validity, as indicated by significant positive correlations with the level of stress experienced during an average week (*r* = 0.39, *p* < 0.001) and the frequency of stressful life events in the past year (*r* = 0.32, *p* < 0.001). Additionally, it exhibited adequate convergent validity, as evidenced by its associations with measures of both physical and mental health. The developers endorsed the use of the PSS-10 in future research due to its comparable psychometric properties with the original version. Furthermore, subsequent studies consistently reaffirmed these findings (25–27).

The scale consists of 4 positively worded items (4, 5, 7, and 8) and 6 negatively worded items (1, 2, 3, 6, 9, and 10), which are rated on a 5-point Likert scale (0 = never, 1 = almost never, 2 = sometimes, 3 = fairly often, or 4 = very often). The total score ranges from 0 to 40, with scores of 0–13, 14–26, and 27–40 indicating low, moderate, and high levels of stress, respectively (28, 29).

Seven Source of Stress items were added, rated on a 5-point Likert scale (0–4; 0 = not at all stressful, 4 = majorly stressful), to capture responses related to the PSS. These items were designed to assess the significance of each source of stress, including work environment, patient care, personal safety, home life, social isolation, financial toxicity, and having children at home due to school closures.

In our study, we employed both the validated English version (30) and the Arabic versions (31, 32) of the questionnaire.

#### 2.3.2 Connor-Davidson Resilience Scale (CD-RISC-10)

The CD-RISC-10 is a validated self-reported tool used to measure resilience in individuals with post-traumatic stress disorder (PTSD) or other types of anxiety. Derived from the CD-RISC-25 (33), a 25-item scale gauging the ability to cope with adversity, respondents rate items on a five-point scale ranging from 0 (not true at all) to 4 (true nearly all the time) reflecting their feelings and experiences over the course of the past month. The total score ranges from 0 to 40 with higher scores reflecting greater resilience. Preliminary investigations into the psychometric properties of the CD-RISC in both the general population and patient samples have substantiated its internal consistency, test–retest reliability, and convergent and divergent validity (33). In a study conducted by Campbell-Sills and Stein (34), the psychometric properties of the CD-RISC-25 were examined. Their research identified an unstable factor structure in the CD-RISC across two equivalent samples via exploratory factor analysis (EFA). Following a series of modifications, a 10-item unidimensional scale was developed, exhibiting robust internal consistency and construct validity. Multiple study’s results underscore the excellent psychometric qualities of the 10-item version, affirming its reliability as a tool for assessing resilience (35–37). The new version uses a five-point scale (0–4). The total score ranges from 0 to 40, with score ranges of 0–29, 30–32, 33–36, and 37–40, indicating lower to higher levels of resilience, with the median score for the general population residing at 32 (38). In our study, we employed the original English version (34) and the validated Arabic version (39) of the questionnaire.

#### 2.3.3 Moral Distress Thermometer (MDT)

Moral distress refers to distress experienced when individuals know the morally right thing to do but are unable to do so due to perceived obligations (40, 41). In their study, Wocial and Weaver (42) initially developed and assessed the psychometric properties of a novel tool designed for detecting moral distress, referred to as the Moral Distress Thermometer (MDT). In their cross-sectional investigation, they presented compelling evidence supporting the MDT’s validity as a subjective measure of moral distress among HCPs when compared to the more comprehensive Moral Distress Scale (MDS) 2009. The validation process of the MDT involved its comparison with the more extensive Moral Distress Scale (MDS) 2009, a tool specifically designed for assessing moral issues (43). The MDT utilizes an 11-point scale, spanning from 0 to 10, along with associated verbal descriptors. Respondents are required to rate the level of moral distress they have experienced in relation to their work over the past 2 weeks. The study findings indicated a moderate correlation between the MDT and the MDS 2009, thereby confirming convergent validity. Concurrent validity was established by comparing MDT scores among different groups of nurses based on their experiences with leaving positions due to moral distress, and these results were consistent with the findings obtained using the MDS 2009. Given the dynamic and subjective nature of moral distress, the authors determined that reliability testing for the MDT was impractical. In our study, Participants are asked to circle the number that best describes the extent to which they experienced work-related moral distress over the past 2 weeks. A score of 4 and above indicates high moral distress. Both the validated English (42) and Arabic (44) versions were used.

In response to the rapidly evolving pandemic circumstances, we made the decision to shorten the recall period to 7 days (45). This adjustment aimed to capture immediate and contextually relevant stressors and experiences encountered by HCPs in their demanding roles within the healthcare system. Given that the importantly, we maintained this consistent 7-day recall period for HCPs in both the initial and subsequent survey waves. This deliberate approach enabled us to longitudinally evaluate stress level, resilience and moral distress while taking into account the dynamic nature of the pandemic’s impact on HCPs.

#### 2.3.4 Socio-demographic data

Socio-demographic data included gender (male or female), occupation (e.g., doctor, nurse, other HCP), age group (≤ 33, > 33), marital status, having children below the age of 18, workplace (in-patient, out-patient, administration), and health sector (MOH, RMS, Private Sector, University hospitals, KHCC).

### 2.4 Measurement translation

Systematic and culturally sensitive translations of the three validated measurements into Arabic for use in the context of Jordan, This comprehensive process strictly adhered to the EORTC (European Organisation for Research and Treatment of Cancer) translation procedures (46). This involved independent forward translations by two bilingual speakers with End-of-Life Care (EoLC) knowledge, professional back translation, and harmonization of all versions.

Then, we systematically compared our reconciled Arabic version with the Arabic-validated questionnaires for PSS, CD-RISC and MDT. This meticulous comparison revealed that there were no disparities between our translated versions and the established and validated Arabic tools designed for these measurements. Consequently, we decided to utilize the three previously validated Arabic versions of the questionnaires (PSS-10, CD-RISC-10, and MDT).

### 2.5 Data collection procedure

The survey was primarily conducted through an online survey platform to ensure participant anonymity and safety during the pandemic. Participants were provided with secure links to the surveys, which were accessible on various devices. The survey was available in Arabic and English language to accommodate the diverse backgrounds of the participants. The eligible participants were identified and contacted by their managers, who informed them about the voluntary nature of participation and clarified that their decision would not affect their performance evaluation. Subsequently, the participants received an invitation and a survey information letter via their official emails. Interested participants contacted the research team, and those who wished to participate or obtain more information were provided with a survey link via their official emails.

A follow-up assessment was performed on a subsample of participants who agreed to repeat the measurement within 2 months of the initial survey to evaluate changes in their responses to the three validated tools over time.

### 2.6 Ethical considerations

Data collection was carried out in a structured and standardized manner. All participants provided informed consent before participating in the study. And were explicitly informed that their consent was implied by reading the survey information letter and completing the survey. They were also assured that they could withdraw from the study at any time without providing a reason until they submitted their completed questionnaires, after which data withdrawal would not be possible.

Ethical approval for the study protocol, including data collection procedures, was obtained from the Institutional Review Board at the King Hussein Cancer Centre (KHCC) under the reference number EC/Ref No: 20 KHCC 123.

### 2.7 Statistical analysis

#### 2.7.1 Sample size estimation

In this study, we employed the Krejcie and Morgan table (47), a well-established and widely accepted method for estimating survey sample sizes. This approach prescribes a minimum sample size of 384 respondents to ensure adequate representation and randomness when dealing with a population size of 10,000.

#### 2.7.2 Statistics

Data were collected using Survey Monkey and exported to SPSS software (V.26) for analysis. Missing data of no more than 10% were accepted by the research team using the Pairwise deletion approach. The responses were analyzed and summarized, and correlations were run with the answered items, resulting in different sample sizes per item.

The significance level was set at *p* < 0.05. The original scores of the three validated tools were presented as mean with standard deviation. The ranked data derived from the counts of each level for PSS, CD-RISC, and MDT were presented as numbers and percentages, likely indicating the distribution of responses across different levels. Independent *t*-tests and one-way ANOVA tests were used to find associations between different variables such as gender, age group, marital status, professional role, workplace, health sector, and having children below the age of 18. A paired *t*-test was performed in a subsample to evaluate responsiveness to change. Variables that showed significance in the analysis were entered into linear regression model. Adjusted *p*-values were reported, indicating the significance of each variable while controlling for the influence of other variables in the model.

## 3 Results

A total of 1,310 healthcare professionals (HCPs) participated in the survey, with 965 completing the initial survey, resulting in a response rate of 74%. Among these respondents, 340 expressed their willingness to participate in a follow-up survey within 2 months, and 239 completed the second survey.

### 3.1 Participant characteristics

Participant characteristics revealed that the majority of participants were male (763, 79.1%). The mean age was 32.74 years (range: 20–74). Among the participants, 435 (45.1%) were doctors, 314 (32.6%) worked in Ministry of Health Hospitals, 550 (57.1%) were married, 546 (56.6%) had children below the age of 18, and 568 (58.9%) worked in an in-patient care setting (Table 1).

**Table 1:** Participants’ demographic characteristics (*n* = 964).

	1^st^ Survey		2^nd^ Survey
	*N* (%)		*N* (%)
**Gender**			
Male	763 (79.1)		181 (75.7)
Female	201 (20.9)		58 (24.3)
**Professional role**			
Doctors	435 (45.1)		99 (41.9)
Nurses	335 (34.8)		89 (37.7)
Other HCPs	194 (20.1)		48 (20.3)
**Sector**			
MoH	314 (32.6)		74 (31)
RMS	126 (13.1)		22 (9.2)
University hospitals	124 (12.9)		34 (14.2)
Private sector	178 (18.5)		43 (18)
KHCC	222 (23)		56 (23.4)
**Marital status**			
Single	395 (41)		92 (38.5)
Married	550 (57.1)		142 (59.4)
Divorced	15 (1.6)		4 (1.7)
Widowed	4 (0.4)		1 (0.4)
**Having children < 18**			
Yes	546 (56.6)		130 (54.4)
No	418 (43.4)		99 (41.4)
**Workplace**			
In-patient	568 (58.9)		190 (54.5)
Out-patient	327 (33.9)		99 (41.4)
Admin	69 (7.2)		190 (54.5)
**Age**			
Mean (Range)	SD		
32.74 (20–74)	8.052		

MoH indicates Ministry of Health; RMS indicates Royal Medical Services; KHCC indicates King Hussein Cancer Centre; Other HCPs indicates any allied health professional.

### 3.2 Outcome measurements

**Figure 1:**
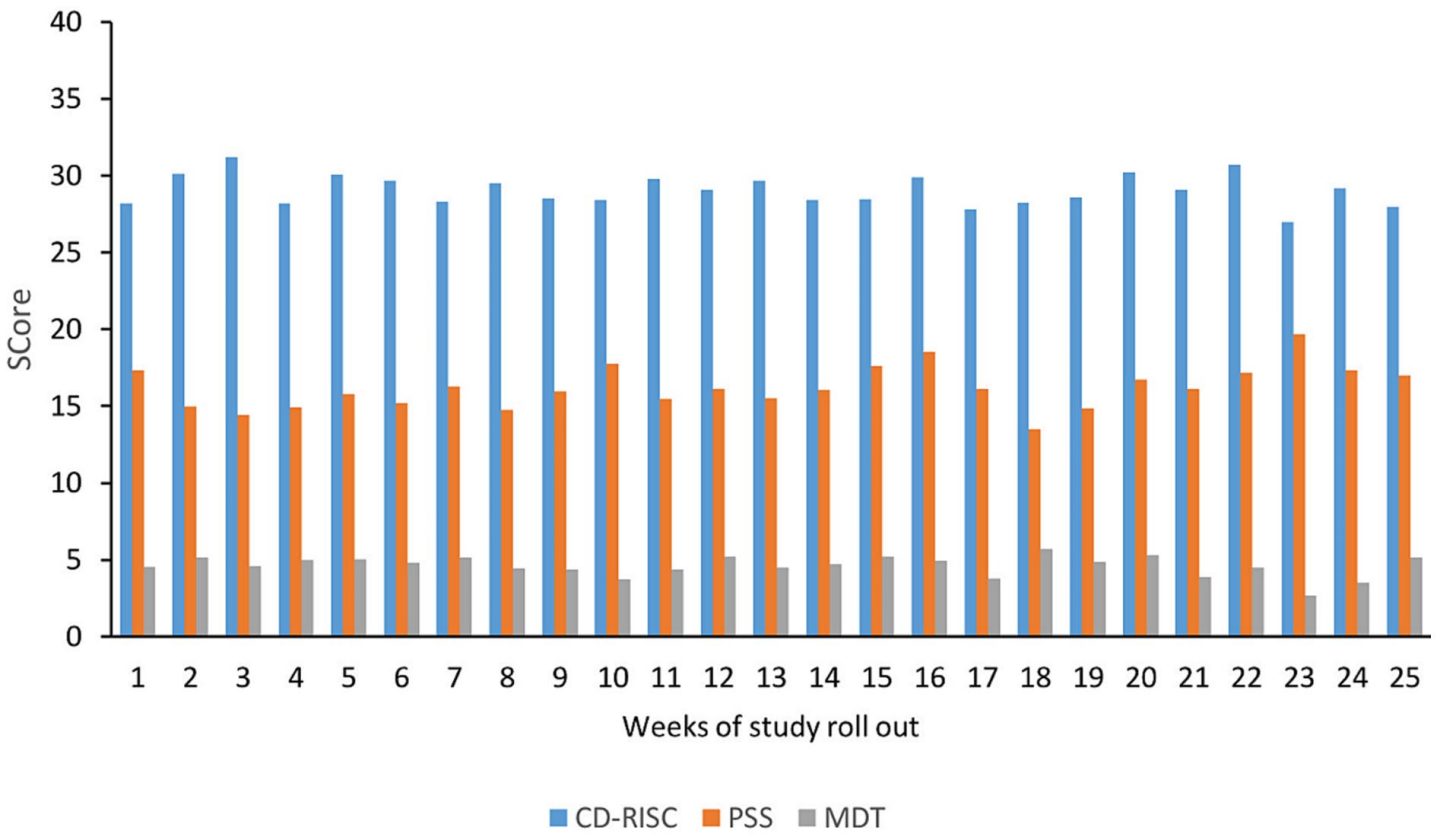
PSS, CD-RISC, and MDT scores in the initial survey: the figure illustrates the Perceived Stress Scale (PSS), Connor-Davidson Resilience Scale (CD-RISC), and Moral Distress Thermometer (MDT) scores on the vertical axis. The horizontal axis represents the week of the study rollout. PSS scores are categorized as low (0–13), moderate (14–26), and high (27–40) levels of stress. CD-RISC scores range from 0 to 29, 30 to 32, 33 to 36, and 37 to 40, with higher scores indicating greater resilience levels. MDT scores range from 0 to 10, with a score of 4 and above indicating high moral distress.

Figure 1 summarizes the scores for the Perceived Stress Scale (PSS), Connor-Davidson Resilience Scale (CD-RSIC), and Moral Distress Thermometer (MDT) over 25 weeks. The mean scores were 15.87 for PSS, 29.18 for resilience, and 4.72 for MDT.

#### 3.2.1 The Perceived Stress Scale (PSS)

The analysis using the Perceived Stress Scale (PSS) indicated that being female (*p* = 0.009), unmarried (*p* = 0.001), having no children below the age of 18 (*p* = 0.000), and being younger (*p* < 0.001) were significantly associated with higher PSS scores. However, occupation (*p* = 0.254), health sector (*p* = 0.637), and workplace (*p* = 0.357) did not have a significant impact on PSS scores. In the summary linear regression analysis involving independent variables and PPS-10 scores, it was observed that gender and age emerged as significant predictors for PPS-10, with *p* = 0.002 and *p* < 0.001, respectively (Table 2).

**Table 2:** Participants’ characteristics with respect to the three validated tools of the first survey.

	PSS			CD-RISC			MDT		
	*N* (%)	Mean ± SD	*P*	*N* (%)	Mean ± SD	*P*	*N* (%)	Mean ± SD	*P*
**Gender**			**0.009**			**0.024**			0.809
Male	746 (79.53)	15.61 ± 5.58		763 (79.15)	29.38 ± 5.14		726 (80.22)	4.71 ± 2.58	
Female	192 (20.47)	16.85 ± 6.78		201 (20.85)	28.45 ± 5.35		179 (19.78)	4.76 ± 2.50	
**Occupation**			0.254			0.494			0.196
Doctors	419 (44.67)	15.94 ± 5.72		435 (45.12)	29.16 ± 5.04		405 (44.75)	4.71 ± 2.51	
Nurses	327 (34.86)	16.13 ± 5.86		335 (34.75)	29.00 ± 5.41		315 (34.81)	4.88 ± 2.51	
Other HCPs	192 (20.47)	15.27 ± 6.15		194 (20.12%)	29.55 ± 5.18		185 (20.44)	4.45 ± 2.76	
**Sector**			0.637			0.114			0.247
MoH	309 (32.94)	15.81 ± 5.72		314 (32.57)	28.88 ± 5.22		302 (33.37)	4.75 ± 2.44	
RMS	119 (12.69)	15.55 ± 5.30		126 (13.07)	28.56 ± 5.33		110 (12.13)	4.55 ± 2.49	
University hospitals	121 (12.90)	15.78 ± 5.73		124 (12.86)	28.98 ± 5.36		114 (12.60)	4.18 ± 2.37	
Private	175 (18.66)	16.48 ± 5.87		178 (18.46)	29.91 ± 4.90		169 (18.67)	4.50 ± 2.80	
KHCC	214 (22.81)	15.68 ± 6.42		222 (23.03)	9.50 ± 5.18		210 (23.2)	4.70 ± 2.66	
**Marital status**			**<0.001**			0.034			0.586
Single	382 (40.72)	16.91 ± 5.76		395 (40.98)	28.78 ± 5.12		365 (40.33)	4.68 ± 2.57	
Married	539 (57.46)	15.06 ± 5.80		550 (57.05)	29.41 ± 5.22		524 (57.9)	4.72 ± 2.57	
Divorced	14 (1.49)	18.64 ± 6.57		15 (1.56)	29.93 ± 5.55		13 (1.44)	5.31 ± 2.43	
Widowed	3 (0.32)	14.67 ± 3.51		4 (0.41)	35.00 ± 3.16		3 (0.33)	6.33 ± 1.53	
**Having children < 18**			**<0.001**			0.44			0.911
Yes	528 (56.29)	15.24 ± 5.81		546 (56.64)	29.30 ± 5.21		512 (56.57)	4.73 ± 2.50	
No	410 (43.71)	16.67 ± 5.84		418 (43.36)	29.04 ± 5.18		393 (43.43)	4.71 ± 2.64	
**Workplace**			0.357			0.907			0.492
In-patient	550 (58.64)	15.83 ± 6.03		568 (58.92)	29.19 ± 5.11		531 (58.67)	4.77 ± 2.50	
Out-patient	322 (34.33)	15.72 ± 5.67		327 (33.92)	29.12 ± 5.35		312 (34.48)	4.69 ± 2.68	
Admin^a^	66 (7.04)	16.85 ± 5.30		69 (7.16)	29.42 ± 5.23		62 (6.85)	4.37 ± 2.50	
**Age group**			**<0.001**			**0.001**			0.517
≤ 33	577 (61.51)	16.72 ± 5.88		594 (61.62)	28.76 ± 5.19		555 (61.33)	4.76 ± 2.55	
> 33	361 (38.49)	14.50 ± 5.58		370 (38.38)	29.86 ± 5.15		350 (38.67)	4.65 ± 2.58	

Significant values are written in bold. *p* ≤ 0.05. MoH indicates Ministry of Health; RMS indicates Royal Medical Services; KHCC indicates King Hussein Cancer Centre; Other HCPs indicates any allied health professional.

a Admin indicates the non-medical staff.

##### 3.2.1.1 Source of stress

The sources of stress were categorized as follows: “Not at all stressful” or “Almost never stressful” (indicating minor stress), “Sometime stressful” (indicating moderate stress), and “Fairly often stressful” or “A major cause of stress” (indicating major stress).

##### 3.2.1.2 Gender

Significant associations were observed between gender and sources of stress. Females reported higher levels of major stress from the imposed social isolation (59.7% vs. 44.5% for males, *p* = 0.000), the risk of contracting COVID-19 (51.6% vs. 39.2% for males, *p* = 0.001), the risk of transmitting COVID-19 to others (77.2% vs. 66.2% for males, *p* = 0.017), and having children home from school (46.2% vs. 29.7% for males, *p* = 0.000). No significant association was found between gender and stress related to providing patient care (*p* = 0.088), the daily work environment (*p* = 0.733), or personal or familial financial concerns (*p* = 0.988) (Table 3).

**Table 3:** Differences between genders and occupation with respect to sources of stress.

	Gender			Occupation			
Category	Male no. (%)	Female no. (%)	*P* value	Doctor no. (%)	Nurse no. (%)	Other HCPs no. (%)	*P* value
Daily work environment							
Minor stress	178 (24.3)	38 (20.7)	0.733	99 (24.0)	60 (18.8)	57 (30.5)	0.001*
Sometimes	297 (40.5)	72 (39.1)		172 (41.7)	117 (36.7)	80 (42.8)	
Major stress	259 (35.3)	74 (40.2)		141 (34.2)	142 (44.5)	50 (26.7)	
Total	734	184		412	319	187	
Providing patient care							
Minor stress	296 (40.3)	61 (33.2)	0.088	132 (32.0)	125 (39.2)	100 (53.5)	<0.001*
Sometimes	280 (38.1)	71 (38.6)		187 (45.4)	112 (35.1)	52 (27.8)	
Major stress	158 (21.5)	52 (28.3)		93 (22.6)	82 (25.7)	35 (18.7)	
Total	734	184		412	319	187	
Risk of becoming infected with COVID-19							
Minor stress	250 (34.1)	41 (22.3)	0.001*	141 (34.3)	91 (28.5)	59 (31.6)	0.301
Sometimes	196 (26.7)	48 (26.1)		108 (26.3)	96 (30.1)	40 (21.4)	
Major Stress	287 (39.2)	95 (51.6)		162 (39.4)	132 (41.4)	88 (47.1)	
Total	733	184		411	319	187	
Concerns of transmitting COVID-19 to others							
Minor Stress	79 (10.8)	15 (8.2)	0.017*	44 (10.7)	28 (8.8)	22 (11.8)	0.186
Sometimes	167 (22.8)	27 (14.7)		96 (23.3)	62 (19.4)	36 (19.3)	
Major Stress	488 (66.5)	142 (77.2)		272 (66.0)	229 (71.8)	129 (69.0)	
Total	734	184		412	319	187	
The need for social isolation							
minor stress	174 (23.7)	25 (13.6)	<0.001*	141 (34.3)	91 (28.5)	59 (31.6)	0.301
Sometimes	233 (31.7)	49 (26.6)		108 (26.3)	96 (30.1)	40 (21.4)	
Major stress	327 (44.6)	110 (59.8)		162 (39.4)	132 (41.4)	88 (47.1)	
Total	734	184		411	319	187	
Personal or family financial concerns							
Minor stress	121 (16.5)	29 (15.8)	0.988	76 (18.5)	50 (15.7)	24 (12.8)	0.065
Sometimes	205 (28.0)	49 (26.6)		125 (30.4)	81 (25.4)	48 (25.7)	
Major stress	407 (55.5)	106 (57.6)		210 (51.1)	188 (58.9)	115 (61.5)	
Total	733	184		411	319	187	
Having children home from school							
Minor stress	395 (53.8)	71 (38.6)	<0.001*	255 (61.9)	125 (39.2)	86 (46.0)	<0.001*
Sometimes	121 (16.5)	28 (15.2)		65 (15.8)	62 (19.4)	22 (11.8)	
Major stress	218 (29.7)	85 (46.2)		92 (22.3)	132 (41.4)	79 (42.2)	
Total	734	184		412	319	187	

Significant values are written in bold. *p ≤ 0.05.

##### 3.2.1.3 Occupation

Profession (doctors, nurses, and other HCPs) also showed a significant correlation with certain sources of stress. Other HCPs reported minor stress from providing patient care more frequently (53.0% vs. 39.2% for nurses and 32.1% for doctors, *p* = 0.000). Nurses demonstrated higher levels of major stress from the daily work environment compared to doctors and other HCPs (44.5% vs. 34.3% for doctors and 26.7% for other HCPs, *p* = 0.001). Other HCPs showed minor stress from the need for social isolation more frequently (55.6% vs. 42.3% for doctors and 49.8% for nurses, *p* = 0.023). Doctors reported minor stress from having children home from school more often (61.9% vs. 46.0% for other HCPs and 39.2% for nurses, *p* = 0.000).

However, the model did not show a significant association between occupation and other sources of stress, such as the risk of contracting COVID-19 (*p* = 0.301), transmitting COVID-19 to others (*p* = 0.186), or personal or familial financial concerns (*p* = 0.065) (Table 3).

#### 3.2.2 The Connor-Davidson Resilience Scale (CD-RISC)

Resilience significantly decreased in females (*p* = 0.024), unmarried individuals (*p* = 0.034), and those of younger age (*p* = 0.001). However, occupation (*p* = 0.494), health sector (*p* = 0.114), having children below the age of 18 (*p* = 0.440), and workplace (*p* = 0.907) did not show any significant effects on resilience. In the summary of the linear regression analysis that incorporated independent variables alongside CD-RISC-10 scores, both gender and age emerged as statistically significant predictors for CD-RISC-10, with *p*-values of 0.01 and *p* = 0.020, respectively (Table 2).

#### 3.2.3 The Moral Distress Thermometer (MDT)

Throughout the entire survey period of 25 weeks, no statistically significant relationship was observed between the participants’ demographic characteristics and the levels of perceived moral distress (Table 2).

#### 3.2.4 Paired data

**Figure 2:**
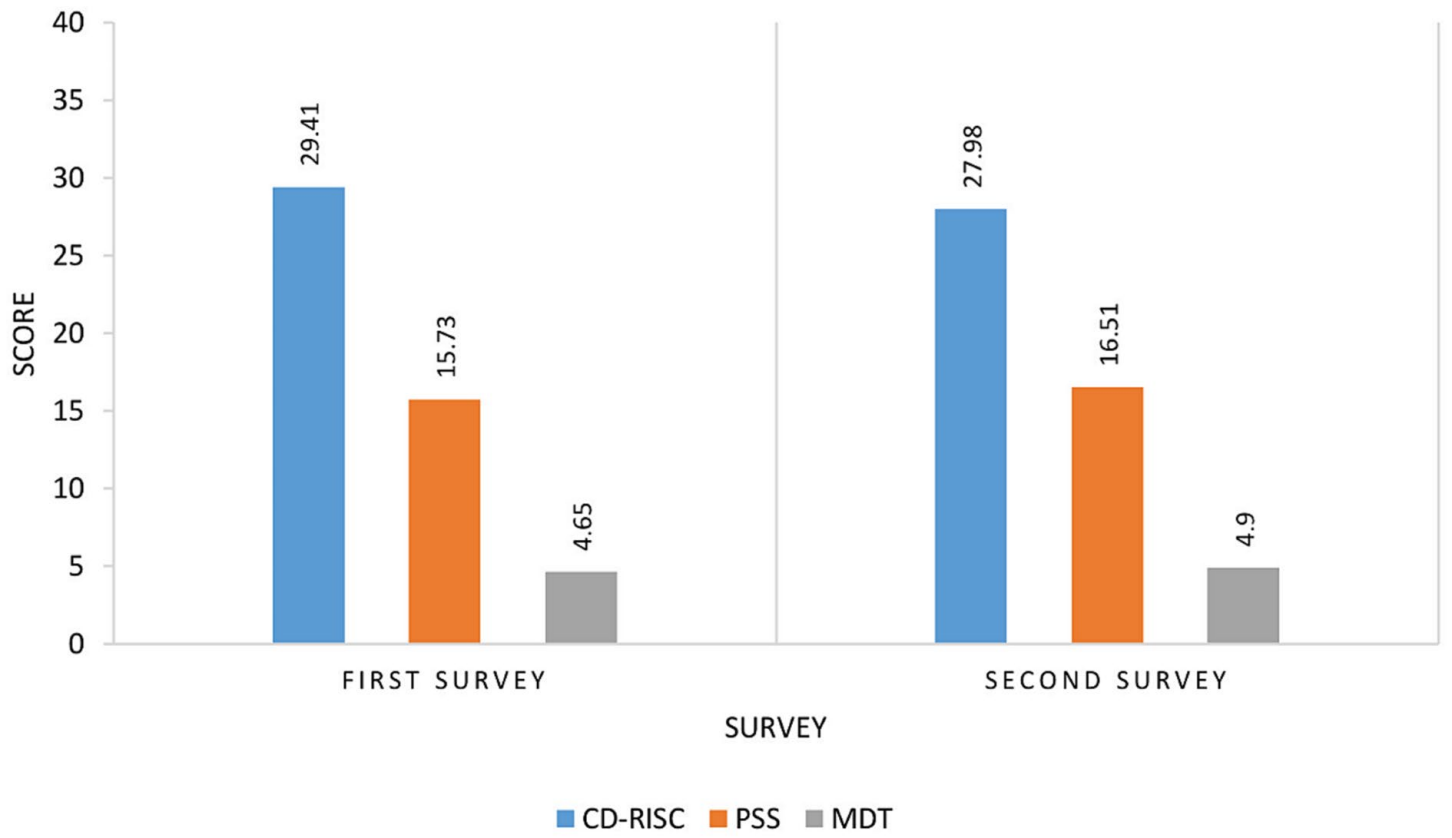
Comparison of CD-RISC, PSS, and MDT between Paired Sample (*n* = 239). PSS, Perceived Stress Scale; CD-RISC, Connor-Davidson Resilience Scale; MOT, The Moral Distress Thermometer. The scores of CD-RISC, PSS, and MDT are shown on the vertical line, and the horizontal line represents the paired surveys during the study roll-out. The PSS scores range from 0 to 13, 14 to 26, and 27 to 40, indicating low, moderate, and high stress levels, respectively. The CD-RISC scores range from 0 to 29, 30 to 32, 33 to 36, and 37 to 40, with a higher score indicating higher resilience. The MDT scores range from 0 to 10, with a score of 4 and above indicating high moral distress.

The statistical model revealed a significant decline only in the CD-RISC scores (*p* < 0.001) for participants in the second survey, while other outcomes remained unaffected (Figure 2).

## 4 Discussion

In the midst of the COVID-19 pandemic, healthcare professionals (HCPs) have faced unprecedented challenges that have significantly impacted their mental well-being. The present study aimed to assess the impact of the COVID-19 pandemic on the stress, resilience, and moral distress levels of healthcare professionals (HCPs) in Jordan over a period of 25 weeks.

The findings of the study indicate that oncology HCPs in Jordan reported moderate levels of perceived stress, low resilience, and high levels of perceived moral distress. These results are consistent with previous studies conducted during the COVID-19 pandemic (11, 12, 48, 49). The waves and peaks of the pandemic in Jordan between 2020 and 2021 likely contributed to the sustained levels of stress and distress experienced by the participants (50).

The Perceived Stress Scale (PSS) scores indicated that being female (22), unmarried (51–57), having no children below the age of 18 (51, 54), and being younger (56, 58–62) were significantly associated with higher stress levels. This aligns with previous research that has shown females and younger individuals experiencing higher stress levels during the pandemic due to factors like increased caregiving responsibilities, work demands, and concerns about personal health and safety (22, 54, 63–65). However, the lack of significant association between occupation groups, health sector, and workplace with stress scores is surprising (52, 55, 66–69). Indicating that the pandemic’s stressors were pervasive across various healthcare professions and settings (66). These findings suggest that the mental well-being of all HCPs should be prioritized, irrespective of their specific roles, and targeted interventions should be implemented to provide adequate support (22).

The sources of stress reported by the participants also showed interesting patterns. Females experienced higher stress levels related to social isolation, the risk of contracting and transmitting COVID-19 (13, 22, 54, 70–73), and having children home from school. This could be attributed to gender-specific societal expectations, such as women shouldering more responsibilities related to caregiving and household tasks during the pandemic (13, 22, 70, 71, 74).

However, stress related to patient care, the daily work environment, and financial concerns was not differentiated by gender (54).

Occupation was also linked to specific stressors such as patient care, the work environment, social isolation, and having children at home (54). While other HCPs reported more stress from providing patient care, Frontline healthcare professionals such as nurses experienced higher stress from the daily work environment, which could be linked to their direct patient care responsibilities and exposure to stressful healthcare settings (13, 57, 75–77). These findings shed light on the unique stressors faced by nurses during the pandemic. Addressing these stressors through targeted interventions, such as mental health support and flexible work arrangements, may be essential in reducing their stress levels and improving overall well-being (13). Although the results did not indicate a statistically significant difference in the Perceived Stress Scale (PSS) scores among HCPs groups regarding stress from personal or family financial concerns, it is worth mentioning that a considerable proportion of them rated it as a major source of stress. This contrasts with the findings of a recent Italian study (54). Its likely influenced by the negative economic impact on families and the national economy (78). The finding that doctors reported more stress from having children home from school is surprising and warrants further investigation. These findings underscore the importance of recognizing the distinct challenges faced by different healthcare professionals and customizing support mechanisms accordingly.

Resilience, as measured by the Connor-Davidson Resilience Scale (CD-RISC), although the participants reported low resilience levels in both surveys, which were below the average resilience score of the general population (38). The decline in resilience scores for participants in the second survey is a noteworthy finding. It suggests that prolonged exposure to the challenges of the pandemic may have eroded participants’ resilience over time, potentially leaving them more vulnerable to stress and burnout (11, 54). The lack of significant changes in stress and moral distress levels may indicate that these factors are relatively stable over the 25-week period or that other external factors such as direct patient care, and the absence of effective vaccines or drugs during the study period may have contributed to a decrease in resilience among HCPs (79).

The Connor-Davidson Resilience Scale (CD-RISC) also showed a significant decline in females, unmarried individuals, and younger participants (56, 58–62). This suggests that certain demographic groups may be more vulnerable to decreased resilience during the pandemic, which could have implications for their mental health and coping abilities (80).

Contrary to some studies, no significant relationship was found between the participants’ demographic characteristics and perceived moral distress levels (52, 55, 67–69), indicating that moral distress may be a universal experience among healthcare professionals during the pandemic, irrespective of their individual characteristics. This highlights the collective challenges faced by healthcare professionals in navigating ethical dilemmas and moral concerns during times of crisis (67–69).

The longitudinal analysis highlighted a significant decline in resilience scores over time. This finding emphasizes the need for continuous mental health support and interventions to sustain HCPs’ well-being throughout the prolonged duration of the pandemic. Implementing resilience-building programs and providing resources for stress management can aid in mitigating potential long-term effects on HCPs’ mental health.

Furthermore, the lack of significant changes in stress and moral distress levels over the two-time point test may indicate that these factors are relatively stable over the 25-week period or that other external factors are compensating for the effects of the pandemic on these outcomes.

## 5 Strengths and limitations

The study had several strengths. First, it included a substantial sample size of 965 healthcare professionals, which enhances the representativeness of the findings. Second, a longitudinal design was employed, allowing for the examination of changes in perceived stress, resilience, and moral distress over time, providing valuable insights into the evolving psychological responses during the pandemic. Third, validated tools were used to measure perceived stress, resilience, and moral distress, increasing the reliability and validity of the study findings.

However, there were also limitations to consider. The initial survey had a cross-sectional design, limiting the ability to establish causal relationships and providing only a snapshot of participants’ psychological responses. Self-report measures were utilized, which may be influenced by response biases such as social desirability bias, potentially affecting the accuracy of reported levels of stress, resilience, and moral distress. The study focused on healthcare professionals in COVID-19-designated hospitals in Jordan, potentially limiting the generalizability of the findings to other healthcare settings or regions. The absence of a control group of healthcare professionals not directly involved in COVID-19 care prevented comparisons and a deeper understanding of the unique stressors and psychological responses associated with the pandemic.

A another limitation of this study is the skewed gender representation, with the majority of participants being male. The low female participation rate may be attributed to cultural and religious factors in Jordanian society, where females may be reluctant to participate in surveys involving direct communication with unrelated males due to cultural norms and preferences for female respondents (81, 82).

Despite these limitations, the study provides valuable insights into the psychological well-being of healthcare professionals during the COVID-19 pandemic. The findings contribute to the existing literature on the subject and highlight the importance of targeted interventions and support strategies to address stress, enhance resilience, and mitigate moral distress among healthcare professionals in similar contexts.

## 6 Conclusion

This study sheds light on the impact of the COVID-19 pandemic on the stress, resilience, and moral distress levels of healthcare professionals in Jordan. The findings highlight the need for targeted support and interventions for vulnerable groups, such as females, unmarried individuals, and younger professionals, to bolster their resilience and mitigate the negative effects of prolonged stress. Healthcare organizations and policymakers should be aware of the sources of stress experienced by different occupational groups to tailor support measures accordingly and promote the well-being of their frontline workers.

## Data availability statement

The raw data supporting the conclusions of this article will be made available by the authors, without undue reservation.

## Ethics statement

The studies involving humans were approved by King Hussein Cancer Center Institutional Review Board. The studies were conducted in accordance with the local legislation and institutional requirements. The ethics committee/institutional review board waived the requirement of written informed consent for participation from the participants or the participants’ legal guardians/next of kin because the survey was conducted over Survey Monkey.

## Author contributions

WA: Conceptualization, Data curation, Funding acquisition, Methodology, Project administration, Supervision, Writing – original draft, Writing – review & editing, Formal analysis. GA: Conceptualization, Data curation, Formal analysis, Funding acquisition, Methodology, Project administration, Supervision, Writing – original draft, Writing – review & editing. KA: Formal analysis, Writing – review & editing. AS: Writing – review & editing. RH: Conceptualization, Methodology, Writing – review & editing. CB: Conceptualization, Methodology, Writing – review & editing. RS: Conceptualization, Methodology, Writing – review & editing. MA-r: Project administration, Resources, Validation, Writing – review & editing. AM: Project administration, Resources, Validation, Writing – review & editing. OS: Conceptualization, Formal analysis, Funding acquisition, Methodology, Project administration, Supervision, Writing – review & editing.
